# Improving the adversarial transferability with relational graphs ensemble adversarial attack

**DOI:** 10.3389/fnins.2022.1094795

**Published:** 2023-02-01

**Authors:** Jiatian Pi, Chaoyang Luo, Fen Xia, Ning Jiang, Haiying Wu, Zhiyou Wu

**Affiliations:** ^1^National Center for Applied Mathematics in Chongqing, Chongqing, China; ^2^Department of Mathematical Sciences, Chongqing Normal University, Chongqing, China; ^3^Mashang Consumer Finance Co., Ltd., Chongqing, China

**Keywords:** multi-model ensemble attack, multi-objective optimization, deep facial recognition, adversarial transferability, graphs

## Abstract

In transferable black-box attacks, adversarial samples remain adversarial across multiple models and are more likely to attack unknown models. From this view, acquiring and exploiting multiple models is the key to improving transferability. For exploiting multiple models, existing approaches concentrate on differences among models but ignore the underlying complex dependencies. This exacerbates the issue of unbalanced and inadequate attacks on multiple models. To this problem, this paper proposes a novel approach, called Relational Graph Ensemble Attack (RGEA), to exploit the dependencies among multiple models. Specifically, we redefine the multi-model ensemble attack as a multi-objective optimization and create a sub-optimization problem to compute the optimal attack direction, but there are serious time-consuming problems. For this time-consuming problem, we define the vector representation of the model, extract the dependency matrix, and then equivalently simplify the sub-optimization problem by utilizing the dependency matrix. Finaly, we theoretically extend to investigate the connection between RGEA and the traditional multiple gradient descent algorithm (MGDA). Notably, combining RGEA further enhances the transferability of existing gradient-based attacks. The experiments using ten normal training models and ten defensive models on the labeled face in the wild (LFW) dataset demonstrate that RGEA improves the success rate of white-box attacks and further boosts the transferability of black-box attacks.

## 1. Introduction

The graph embedding model (Scarselli et al., 2009; Cui et al., 2019) demonstrates the expressive potential of deep learning on graph-structured data, and has shown promise in several applications including the classification of structural roles (Tu et al., 2018), biological analysis (Hamilton et al., 2017), financial monitoring (Paranjape et al., 2017), and the prediction of molecular features (Duvenaud et al., 2015). However, recent research (Dai et al., 2018; Zügner et al., 2018; Bojchevski and Günnemann, 2019) reveals that numerous types of graph embedding techniques, such as Graph Convolutional Networks, DeepWalk, etc., are susceptible to adversarial attacks. Therefore, A lot of attention has been paid to generating adversarial examples, called adversarial attacks, because they may be used to estimate the robustness of various models (Rauber et al., 2020; Tramer et al., 2020) and boost their robustness through adversarial training (Xu et al., 2017; Kurakin et al., 2018b; Mehrabi et al., 2021).

Additionally, adversarial examples often exhibit good transferability across the models (Liu et al., 2016; Papernot et al., 2016; Dong et al., 2019; Chen et al., 2021), i.e., examples created for one model can still deceive other models. Several attack techniques (Szegedy et al., 2013; Carlini and Wagner, 2017; Kurakin et al., 2018b; Madry et al., 2018) treat the adversarial example generation process as an iterative optimization and exhibit high attack performance in a white-box setting (Goodfellow et al., 2014). Nevertheless, in an unknown black-box environment (Papernot et al., 2016), these approaches suffer from a serious lack of transferability.

Previous studies (Dong et al., 2018, 2019; Lin et al., 2019; Xie et al., 2019) attributed the lack of transferability to overfitting surrogate models. Therefore, various techniques have been proposed to mitigate overfitting, including advanced gradient methods (Dong et al., 2018; Lin et al., 2019; Wang and He, 2021; Zou et al., 2022), ensemble multi-model attacks (Liu et al., 2016; Dong et al., 2018; Li et al., 2020), input transformations (Dong et al., 2019; Xie et al., 2019; Wang X. et al., 2021), and model-specific methods (Wu et al., 2019; Guo et al., 2020). Almost experiments (Dong et al., 2018; Zhao et al., 2021) and top entries in competitions (Kurakin et al., 2018a) shows that ensemble multi-model attacks and input transformations (e.g., random resizing and padding, transformations, scaling, etc.) are among the most effective methods. Moreover, Lin et al. (2019) suggested that the input transformation can be conceived as a model augmentation to attack more models. As well, Li et al. (2020) suggested dynamic erosion of certain intermediate structures of the surrogate model to the same end. In conclusion, acquiring and utilizing multiple models is key to achieving better transferability, yet investigations on utilizing multiple models are quite lacking.

For utilizing multiple models, we find that, compared to the two-by-two orthogonality among classification models, facial recognition models have more complex relationships among models. Recall that some real-world tasks, such as recommendation (Fan et al., 2019; Tan et al., 2020), urban data mining (Dai et al., 2020; Wang et al., 2020), and multi-task learning (Chen et al., 2019; Cao et al., 2022), enhance the utilization of information by using graphical representations to capture and exploit pairwise dependent relationships. This perspective leads us to consider the following question: Can we enhance the utilization of multiple models by capturing and exploiting the dependent relationships among the models?

For this purpose, we propose a novel method, called the Relational Graphs Ensemble Attack (RGEA), to improve the transferability. Specifically, 1) To exploit the complex dependencies among models, we redefine the multi-model ensemble attack as multi-objective optimization, and construct Sub-optimization problem 1 to find the optimal attack direction in the iteration; 2) Since the high dimensionality of the images causes a serious time-consuming problem. To eliminates the time-consuming problem, we define the vector representation of the model and the dependency relationships among the models, and build the equivalent Sub-optimization problem 2. 3) Furthermore, we theoretically prove the equivalence between the Sub-optimization problem 1 and 2, as well as analyze the association between RGEA and MGDA (Désidéri, 2012). 4) Extensive experiments on the LFW facial dataset show that RGEA improves the benchmarking methods in a black-box setting, which indicates that RGEA can effectively exploit the dependencies among models to reliably improve transferability.

The remainder of this paper is organized as follows: Section 2 summarizes the related work. Section 3 describes RGEA in terms of motivation, details, and theoretical analysis. Section 4 reports the experimental results and comparisons. Section 5 gives some concluding remarks.

## 2. Related work

In this paper, we choose the more challenging targeted attack, as well as consider the widely studied perturbation constraint of the infinite norm *L*_*inf*_ = ||·||_*inf*_.

### 2.1. Optimization model of adversarial attacks

Suppose {*F*_*i*_(*x*)|*i* = 1, ⋯ , *m*} is a set of pre-trained deep facial recognition models and the corresponding loss function:


(1) $$\begin{array}{l} Loss_{i}\left(x,x_{target}\right)=cos\left(F_{i}\left(x\right),F_{i}\left(x_{target}\right)\right), \end{array}$$

where *x* is the input facial image, *x*_*target*_ is the corresponding target facial image, {*F*_*i*_(*x*)|*i* = 1, ⋯ , *k*} is surrogate models being attacked, and {*F*_*i*_(*x*)|*i* = *k*+1, ⋯ , *m*} is unknown black-box models being tested.

For an arbitrary facial example *x* and a target face *x*_*taget*_, we find the corresponding adversarial example *x*_*adv*_, i.e., maximizes the objective $\sum_{i=1}^{k}Loss_{i}\left(x_{adv},x_{target}\right)$ on the surrogate models, but still keeping the ϵ imperceptibility constraint, as the following constrained optimization problem:


(2) $$\begin{array}{l} x_{adv}=\underset{x_{adv}:||x_{adv}-x|{|}_{inf}}{argmax}\;\overset{k}{\underset{i=1}{\sum }}Loss_{i}\left(x_{adv},x_{target}\right) \end{array}$$

### 2.2. The gradient-based methods

In this section, we introduce a series of gradient-based methods which have been developed to improve transferability. Iterative Fast Gradient Sign Method (I-FGSM) (Kurakin et al., 2018b; Madry et al., 2018) was used as the backbone of the gradient methods with *L*_*inf*_ bounded. This iterative approach, given an input *x* and the corresponding target *x*_*target*_, calculates the perturbation output *x*_*T*_ by applying *T* steps of the following updated steps (with *x*_0_ = *x*):


(3) $$\begin{array}{l} x_{t+1}=\prod_{B_{inf}\left(x,\varepsilon \right)}\left(x_{t}+\alpha s_{t}\right),\;s_{t}=\prod_{\partial B_{inf}\left(0,1\right)}\left(\nabla_{x_{t}}Loss\left(x_{t},x_{target}\right)\right), \end{array}$$

Where ∏_*s*_ is the projection into the set *S*, *B*_*inf*_(*x*, ε) is the *L*_*inf*_ ball of radius ϵ around *x*, α is the step size, ∂*U* is the boundary of a set *U*, and *s*_*t*_ is the maximum inner product projection of the gradient ∇_*x*_*t*__*Loss*(*x*_*t*_, *x*_*target*_) at *x*_*t*_ onto the unit *L*_*inf*_ ball.

Since (Goodfellow et al., 2014) proposes that DNNs have linear properties, *s*_*t*_ can be interpreted as the maximum inner product projection of ∇_*x*_*t*__*Loss*(*x*_*t*_, *x*_*target*_) in the local region to enhance the attack ability of adversarial samples after a finite number of iterative attacks. Note that, in the case of the *L*_*inf*_ norm, We can convert (Equation 3) to the following form:


(4) $$\begin{array}{l} x_{t+1}=Clip_{\varepsilon }^{x}\left(x_{t}+\alpha \cdot sign\left(\nabla_{x_{t}}Loss\left(x_{t},x_{target}\right)\right)\right). \end{array}$$

Momentum Iterative Method (MIM) (Dong et al., 2018) improves the transferability by introducing momentum terms in the attack process, given as:


(5) $$\begin{array}{l} m_{t}=\mu \cdot m_{t-1}+\frac{\nabla_{x_{t}}Loss\left(x_{t},x_{target}\right)}{||\nabla_{x_{t}}Loss\left(x_{t},x_{target}\right)|{|}_{1}},\\ x_{t+1}=Clip_{\varepsilon }^{x}\left(x_{t}+\alpha \cdot sign\left(m_{t}\right)\right), \end{array}$$

Where *m*_*t*_ denotes the accumulated gradient at (*t*)_*th*_ iteration, and μ is the decay factor.

The Nesterov accelerated gradient (NIM) (Lin et al., 2019) is integrated into the I-FGSM-based attack method to improve the sensitivity of the momentum method when the current gradient has a large gap in the direction of momentum, and further increases the transferability of adversarial examples, given as:


(6) $$\begin{array}{l} x_{t}^{nes}=x_{t}+\alpha \cdot \mu \cdot m_{t},\\ m_{t}=\mu \cdot m_{t-1}+\frac{\nabla_{x_{t}^{nes}}Loss\left(x_{t}^{nes},x_{target}\right)}{||\nabla_{x_{t}^{nes}}Loss\left(x_{t}^{nes},x_{target}\right)|{|}_{1}},\\ x_{t+1}=Clip_{\varepsilon }^{x}\left(x_{t}+\alpha \cdot sign\left(m_{t}\right)\right). \end{array}$$

Scale-Invariant Method (SIM) (Lin et al., 2019) uses scale replicates of the input image to further enhance the transferability. However, SIM uses a lot more resources and running time, given as:


(7) $$\begin{array}{l} x_{t+1}=Clip_{\varepsilon }^{x}\left(x_{t}+\alpha \cdot sign\left(\frac{1}{m}\overset{m}{\underset{i=1}{\sum }}\nabla_{x_{t}}Loss\left(\frac{x_{t}}{2^{i}},x_{target}\right)\right)\right). \end{array}$$

The Diversity Input Method (DIM) (Xie et al., 2019) applies random resizing and padding to adversarial examples with probability *p* in each iteration to further improve the transferability of the adversarial examples, given as:


(8) $$\begin{array}{l} x_{t+1}=Clip_{\varepsilon }^{x}\left(x_{t}+\alpha \cdot sign\left(\nabla_{x_{t}}Loss\left(T\left(x_{t},p\right),x_{target}\right)\right)\right), \end{array}$$

where *T*(·, *p*) indicates that the input transformation is performed with probability *p*.

Translation-Invariant Method (TIM) (Dong et al., 2019) generates adversarial examples that are insensitive to the discriminative region of the surrogate models by translation invariance and uses predefined convolution kernels instead of translation operations to improve efficiency, given as:


(9) $$\begin{array}{l} x_{t+1}=Clip_{\varepsilon }^{x}\left(x_{t}+\alpha \cdot sign\left(W⊗\nabla_{x_{t}}Loss\left(x_{t},x_{target}\right)\right)\right), \end{array}$$

where *W* is the pre-defined convolution kernel and ⊗ represents the convolution operation.

In conclusion, current approaches analogize transferability to generalizability and move the strategies for enhancing generalizability to gradient-based attack methods. However, they ignore the complex relationships among models, which limits the transferability of generated adversarial samples. Inspired by multi-relational graphs, we find optimal descent directions by solving sub-optimization problems based on relational graphs in iterative attacks, further improving transferability.

### 2.3. Graph-based modeling

Notably, several recent works use graphs to capture and extract dependencies between entities, enhancing the performance of existing models and algorithms. For example, in traffic prediction, ST-GCN (Yu et al., 2018) and ST-MGCN (Geng et al., 2019) propose to model the dependencies among regions by using graph convolutional networks, and then combine the dependencies to further enhance the model prediction. In anomaly detection, SCAN (Xu et al., 2007) captures and extracts the dependencies among entities through graphs, and then detects anomalous entities by looking for anomalous dependencies among them. Likewise, in multi-task learning, ML-GCN (Chen et al., 2019) proposes capturing and exploring dependencies among multiple labels based on graphs, and combining this dependency to improve recognition performance. In addition, RMTL (Cao et al., 2022) proposes capturing data-data and task-task dependencies by building knowledge graphs that connect data points and tasks, and combining these dependencies to achieve more accurate predictions for new tasks. Motivated by these works, we propose the Relational Graphs Ensemble Attack (RGEA), which combined the dependencies among models to facilitate the exploitation of multiple models. It has not been explored in existing attack methods.

## 3. Methodology

**Figure 1:**
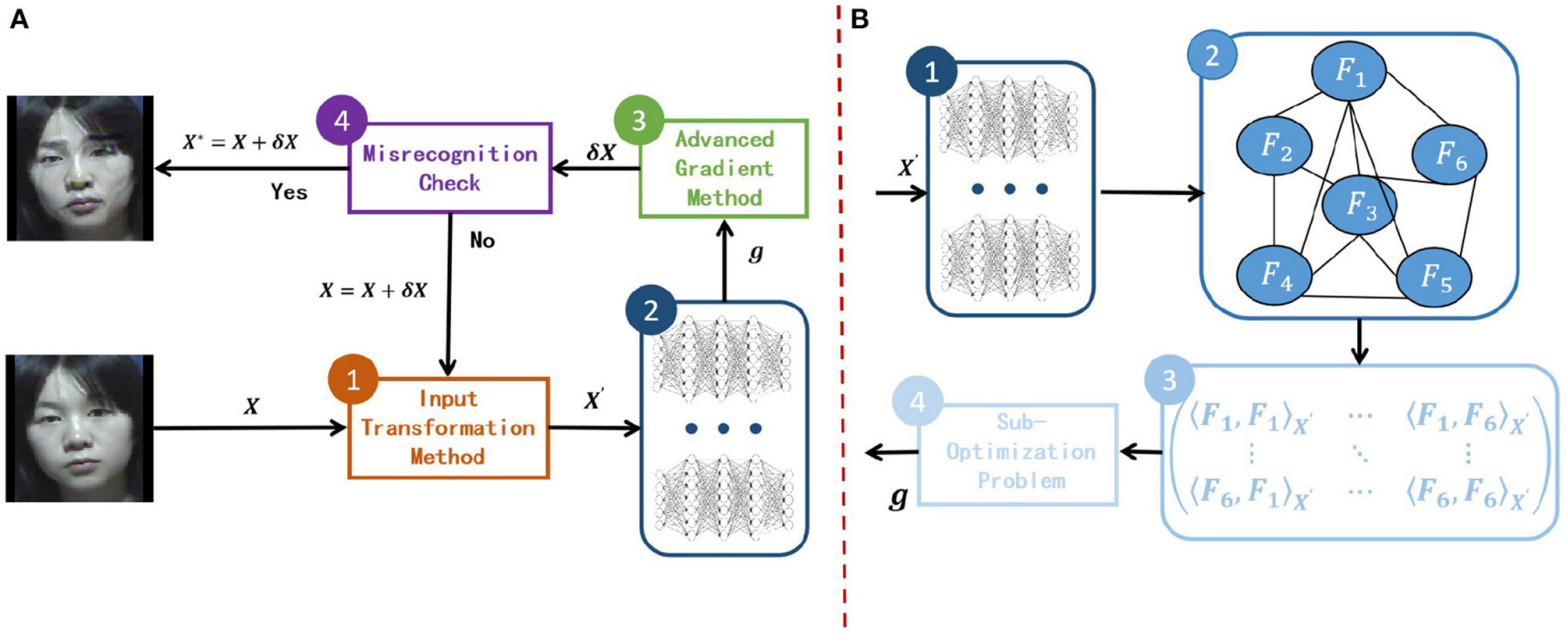
**(A)** An attack framework that combines multi-model ensemble attacks with existing attack methods which are advanced gradient optimization techniques and input transformation techniques. **(B)** The overall RGEA calculation process, where the set of surrogate models is {*F*_*i*_(*x*)|*i* = 1, ⋯ , 6}.

In Section 2.2, we systematically introduce the existing gradient-based methods. Remarkably, combining several methods can further improve the transferability of the adversarial samples. Thus, we give the current general attack framework in Figure 1A by combining multi-model ensemble attacks with advanced gradient methods and input transformations. In this framework, RGEA focuses on the second step, as shown in Figure 1B, where we first construct a multi-model graph; then extract the dependency matrix of the model from the constructed graph; finally, find the final descent direction by constructing a Optimization problem based on the dependency matrix.

Given that our proposed approach is for multi-model ensemble attacks, we first discuss the problems of ensemble attack, then introduce our motivation and elaborate on the proposed RGEA algorithm, and finally provide a comprehensive theoretical analysis of the RGEA algorithm.

### 3.1. The problem of ensemble attack

Intuitively, adversarial examples is more likely to transfer attack capabilities to other models, if it remains an adversary to multiple models. Based on this insight, acquiring and utilizing multiple models is the key to obtaining better transferability. For exploiting multiple models, earlier studies (Dong et al., 2018; Che et al., 2020) focused on uniform fusing the outputs of different layers of DNNs. For example, Liu et al. (2016) pioneered the study of multi-model ensemble attacks and proposed uniform fusion loss, Dong et al. (2018) proposed two uniform fusion strategies for logit or probabilistic outputs of models, and Che et al. (2020) proposed uniform fusion intermediate features. However, they ignore inter-model differences, thereby He et al. (2022) proposed a gradient normalized ensemble attack to address inter-model gradient magnitude differences, and Xiong et al. (2022) proposed a stochastic variance reduction ensemble (SVRE) attack to tackle inter-model gradient variance.

**Figure 2:**
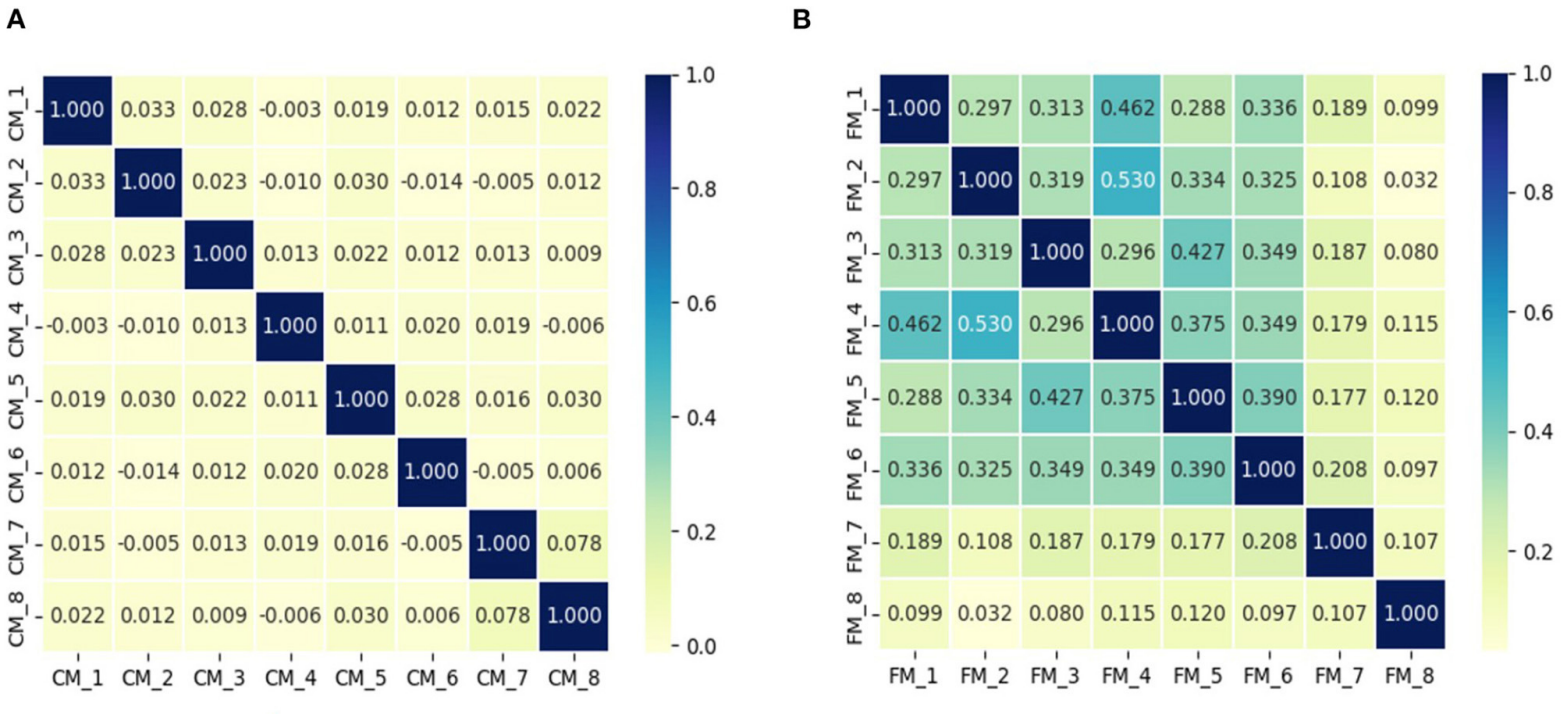
The cosine similarity of the gradients of the sampled photos on the different models is depicted in the figure, where **(A)** is the visualization result of multiple classification models, and **(B)** is the visualization result of multiple deep facial recognition models.

Nevertheless, differences among models only one-sidedly reveal complex dependencies, and we find that the dependencies among deep facial recognition models are more complex than those of classification models. Specifically, Figure 2 shows that multiple deep facial recognition model gradients have larger and more complex cosine similarities, compared to the classification models. In addition, there is a complex inherent similarity between different subsets of surrogate models, as shown in Figure 2B, where the similarity among the top six models is much greater than others. In conclusion, existing approaches only one-sidedly consider the differences among models, and ignore the complex dependencies behind them. It leads to an unbalanced and inadequate attack on multiple models such that it limits transferability black-box attacks. This inspired us to exploit the complex dependencies among models to improve the transferability of the generated adversarial samples.

In essence, existing approaches fail to exploit complex dependencies among models, which stems from their treating multiple models as one complex multivariate model and using single-objective optimization. For this reason, this paper redefines the multi-model ensemble attack as multi-objective optimization, defined as follows:


(10) $$\begin{array}{l} \underset{x_{adv}}{argmax}\;{\left\{J_{i}\left(x_{adv}\right)\right\}}_{i=1,\cdots \;,k},\\ s.t.\;D\left(x,x_{adv}\right)\leq \varepsilon , \end{array}$$

where *J*_*i*_(*x*_*adv*_)∈*R* is a continuous function measuring the attack of the adversarial sample relative to the model *F*_*i*_, usually choosing the loss function *Loss*(*F*_*i*_(*x*_*adv*_), *x*_*target*_). *D*(*x, x*_*adv*_)∈*R* is a continuous function that mainly measures the amount of perturbation in the sample, usually choosing the *L*_*p*_ norm, i.e., ||*x*−*x*_*adv*_||_*p*_, and ϵ is the maximum disturbance distance.

Inspired by MGDA, we performed a theoretical analysis based on the first-order Taylor approximation. Assume that at *x*_*n*_ is the *n* iteration of the adversarial sample, $g_{n}^{*}$ is the final direction of descent, $g_{n}^{i}\left(i=1,\cdots k\right)$ is the gradient of the objective function *J*_*i*_(*x*) computed on *x*_*n*_, and α is the iteration step size. Under the assumptions, we have ${\text{x}}_{\text{n + 1}}={\text{x}}_{n}+\alpha g_{n}^{*}$ and ∇*J*_*i*_(*x*_*n*_) = *J*_*i*_(*x*_*n*+1_)−*J*_*i*_(*x*_*n*_). When the step size is small enough, the following approximate transformations are available:


(11) $$\begin{array}{l} \left(\begin{array}{c} \nabla J_{1}\left(x_{n}\right)\\ \;\cdots \\ \nabla J_{k}\left(x_{n}\right) \end{array}\right)\approx \left(\begin{array}{c} \alpha \langle g_{n}^{1},g_{n}^{*}\rangle \\ \;\cdots \\ \alpha \langle g_{n}^{k},g_{n}^{*}\rangle \end{array}\right)=\alpha ||g_{n}^{*}||\left(\begin{array}{c} ||g_{n}^{1}|{|}_{2}\cos\left(g_{n}^{1},g_{n}^{*}\right)\\ \;\cdots \\ ||g_{n}^{k}|{|}_{2}\cos\left(g_{n}^{k},g_{n}^{*}\right) \end{array}\right), \end{array}$$

Where $\left||g_{n}^{1}||,\cdots \;,||g_{n}^{k}|\right|$ is deterministic and $\left||g_{n}^{*}|\right|$, α is constant, then the effect of each objective's descent in each iteration is primarily influenced by $\cos\left(g_{n}^{1},g_{n}^{*}\right),\cdots \;,\cos\left(g_{n}^{k},g_{n}^{*}\right)$.

In summary, we cam eliminate the problem of unbalanced and inadequate attacks by finding the final descent direction which $cos\left(g_{n}^{1},g_{n}^{*}\right)$ $,\cdots \;,\cos\left(g_{n}^{k},g_{n}^{*}\right)$ is as equal and large as possible. We translate this idea into solving the following optimization problems:


(12) $$\begin{array}{l} {argmin}_{g}\;||g|{|}_{2}\;,\\ s.t.\;\left\{g|{\arg\min}_{g}||Gg-e||,e={\left(1,\cdots \;,1\right)}^{T}\right\}, \end{array}$$

where $G={\left(g_{n}^{1}/||g_{n}^{1}|{|}_{2},\cdots \;,g_{n}^{k}/||g_{n}^{k}|{|}_{2}\right)}^{T}\in R^{k\times N}$ is a Jacobi matrix, *k* is the number of models and *N* is the image dimension. Optimization problem in Equation (12) will be referred to as Optimization problem 1 in the discussion that follows.

### 3.2. Relational graphs ensemble adversarial attack

Since Optimization problem 1 is built based on the high-dimensional space of images, which causes serious time-consuming problems. However, the number of surrogate models employed is much smaller than the image dimension due to the large number of computational resources required to load the models. It stimulates us to explore building a new optimization problem based on the dependencies among the models.

In this paper, we use a graph to extract the dependencies among models, which is a flexible way to capture the topology of the model space. Specifically, we represent each node of the graph as an embedding vector of the model. For better theoretical analysis, we simplify the vector representation and define the embedding vector of model *F*_*i*_ on *x*_*n*_ to be represented as $g_{n}^{i}/||g_{n}^{i}|{|}_{2}$. As well as the dependencies among the models are expressed in the following form:


(13) $$\begin{array}{l} {\langle F_{i},F_{j}\rangle }_{x_{n}}=\cos\left(g_{n}^{i},g_{n}^{j}\right). \end{array}$$

For characteristics of the solution to Optimization problem 1, we have the following proposition.

** Proposition 1**. *If* $g_{n}^{*}$ *the solution of Optimization problem 1, then there exists* w^*^ *such that* $g_{n}^{*}={\text{G}}^{\text{T}}{\text{w}}^{*}$.

Proof. Suppose d_1_⋯ , d_*r*_ is the orthogonal bases of the subspace spaned by *g*_1_⋯ , *g*_*k*_, as well as d_*r*+1_⋯ , d_*n*_ and d_1_⋯ , d_*r*_ are the orthogonal bases of *R*^*n*^. Therefore, there exist $w_{1}^{*}\in R^{r}$ and $w_{2}^{*}\in R^{n-r}$ with $g_{n}^{*}=\left(d_{1},\cdots \;,d_{r}\right)w_{1}^{*}+\left(d_{r+1},\cdots \;,d_{n}\right)w_{2}^{*}$, such that the following equation holds.


(14) $$\begin{array}{l} Gg_{n}^{*}=\text{G}\left({\text{d}}_{1},\cdots \;,d_{r}\right)w_{1}^{*} \end{array}$$

furthermore, Optimization problem 1 takes ||*g*||_2_ to be extremely small, so it follows that $g_{n}^{*}=\left(d_{1},\cdots \;,d_{r}\right)w_{1}^{*}$. From this, there exists w^*^ such that $g_{n}^{*}={\text{G}}^{\text{T}}{\text{w}}^{*}$.

By this proposition, we can denote $g_{n}^{*}$ as a linear combination of each model embedding vector, i.e., $g_{n}^{*}=G^{T}w_{n}$ where *w*_*n*_ is the weight vector of the linear combination, and we can transform the equation in Equation (11) as follows:


(15) $$\begin{array}{lll} \left(\begin{array}{c} \nabla J_{1}\left(x_{n}\right)\\ \;\cdots \\ \nabla J_{k}\left(x_{n}\right) \end{array}\right) & \approx & \left(\begin{array}{c} \alpha \langle g_{n}^{1},g_{n}^{*}\rangle \\ \;\cdots \\ \alpha \langle g_{n}^{k},g_{n}^{*}\rangle \end{array}\right)\\ =\alpha {\left(\begin{array}{ccc} {\langle F_{1},F_{1}\rangle }_{x_{n}} & \ldots & {\langle F_{1},F_{k}\rangle }_{x_{n}}\\ \vdots & \ddots & \vdots \\ {\langle F_{k},F_{1}\rangle }_{x_{n}} & \cdots & {\langle F_{k},F_{k}\rangle }_{x_{n}} \end{array}\right)}_{k\times k}\left(\begin{array}{c} ||g_{n}^{1}|{|}_{2}w_{n}^{1}\\ \;\cdots \\ ||g_{n}^{k}|{|}_{2}w_{n}^{k} \end{array}\right), \end{array}$$

further, associating (Equations 11, 15) and simplifying them yields the following equation:


(16) $$\begin{array}{l} \left(\begin{array}{c} \cos\left(g_{n}^{1},g_{n}^{*}\right)\\ \;\cdots \\ \cos\left(g_{n}^{k},g_{n}^{*}\right) \end{array}\right)=\frac{1}{||g_{n}^{*}|{|}_{2}}{\left(\begin{array}{ccc} {\langle F_{1},F_{1}\rangle }_{x_{n}} & \ldots & {\langle F_{1},F_{k}\rangle }_{x_{n}}\\ \vdots & \ddots & \vdots \\ {\langle F_{k},F_{1}\rangle }_{x_{n}} & \cdots & {\langle F_{k},F_{k}\rangle }_{x_{n}} \end{array}\right)}_{k\times k}\left(\begin{array}{c} w_{n}^{1}\\ \cdots \\ |w_{n}^{k} \end{array}\right), \end{array}$$

hence, we establish the connection between $\cos\left(g_{n}^{1},g_{n}^{*}\right),\cdots \;,\cos\left(g_{n}^{k},g_{n}^{*}\right)$ and dependency among models. With this connection, we can transform Optimization problem 1 as follows, and the proof of equivalence transformation is given in Section 3.3.


(17) $$\begin{array}{l} {argmin}_{w}\;||Aw-e|{|}_{2}\;,\\ whereA=GG^{T}={\left(\begin{array}{ccc} {\langle F_{1},F_{1}\rangle }_{x_{n}} & \ldots & {\langle F_{1},F_{k}\rangle }_{x_{n}}\\ \vdots & \ddots & \vdots \\ {\langle F_{k},F_{1}\rangle }_{x_{n}} & \cdots & {\langle F_{k},F_{k}\rangle }_{x_{n}} \end{array}\right)}_{k\times k},e=\left(1,\cdots \;,1\right), \end{array}$$

where *A* is a multi-relationship matrix transformed from a multi-model diagram, and the ultimate direction of descent $g_{n}^{*}$ is equal to $G^{T}w_{n}^{*}$, where $w_{n}^{*}$ is the solution to the optimization issue in Equation (17). Optimization problem in Equation (17) will be referred to as Optimization problem 2 in the discussion that follows.

Theoretically, RGEA can be used with a variety of currently used iterative gradient-based attack strategies. An explanation of the integration of RGEA and I-FGSM is provided by Algorithm 1. Because the descent direction determined by solving optimization problem 2 maintains the optimal angle to multiple objective gradients. In Algorithm 1, we replace the symbolic operation of the I-FGSM algorithm with the normalization method as follows:


(18) $$\begin{array}{l} g^{*}=\frac{g^{*}\cdot \sqrt{N}}{||g^{*}|{|}_{2}},\;where\;g\in R^{N}. \end{array}$$

**Algorithm 1:** The RGEA-I-FGSM attack algorithm

**Input:** A benign example *x* and its label *y*, a set of *k* surrogate models, corresponding losses {*J*_1_, ⋯ , *J*_*k*_}, the perturbation bound ϵ, number of iterations *T*, and the iteration step α.
**Output:** An adversarial example *x*^*adv*^ that fulfills $||x^{adv}-x|{|}_{\infty }$
1: Initialize $x_{0}^{adv}=x$;
2: **for** *t* = 0 to *T*−1 **do**
3: Get the loss of the models $\left\{J_{1}\left(x_{t}^{adv}\right),\cdots \;,J_{k}\left(x_{t}^{adv}\right)\right\}$;
4: Calculate the gradient of the models $\left\{g_{t}^{1},\cdots \;,g_{t}^{k}\right\}$:	$g_{t}^{i}=\nabla_{x_{t}^{adv}}J\left(x_{t}^{adv},y\right);$
5: Get the Jacobi matrix of multiple objectives:	$G={\left({\text{g}}_{t}^{1},\cdots \;,{\text{g}}_{t}^{k}\right)}^{T};$
6: Calculate the solution of Optimization problem 2 $w_{t}^{*}$:	$$w_{t}^{*}={\arg\min}_{w}||Aw-{\left(1,\cdots \;,1\right)}^{T}||;$$
7: Calculating the final direction of descent $g_{t}^{*}$:	$$g_{t}^{*}=G^{T}w_{t}^{*};$$
8: Update $x_{t+1}^{adv}=Clip_{x}^{\varepsilon }\left\{x_{t}^{adv}+\alpha \cdot \frac{g_{t}^{*}\cdot \sqrt{N}}{||g_{t}^{*}|{|}_{2}}\right\}$
9: **end for**
10: **return** $x^{adv}=x_{T}^{adv}$

We perform ablation experiments on this in Section 4.2.2 to investigate the effect of this operation on the transferability of the generated adversarial samples.

### 3.3. Theoretical analysis of RGEA

According to Section 3.2, the key core of RGEA is Optimization problem 2, and we next present a detailed theoretical analysis of Optimization problem 2. We first demonstrate that Optimization Problems 2 and 1 are equivalent, after which we discuss the relationship between MGDA and RGEA.

Continuing with the notation in Section 3.2, we next state the proposition on the equivalence between Optimization problem 2 and Optimization problem 1, and proof it.

** Proposition 2**. *The assumption is that* *G*∈*R*^*k*×*N*^ *is a matrix and* *A* = *GG*^*T*^ *is a matrix belonging to* *R*^*k*×*k*^*. Then for arbitrary* *b, there is an equivalence between Optimization problem 2 and Optimization problem 1*.

Proof. Let A^*^ and G^*^ be the Moore-Penrose generalized inverse matrices of *A* and *G*, respectively, then we get the following equation:


(19) $$\begin{array}{l} G^{*}GG^{*}=G^{*},GG^{*}G=G,{\left(GG^{*}\right)}^{T}=GG^{*},{\left(G^{*}G\right)}^{T}\\ =G^{*}G,A^{*}={G^{*}}^{T}G^{*}. \end{array}$$

Given that A^*^ is the Moore-Penrose generalized inverse matrices, the general solution to Optimization problem 2 can be formulated as w^*^ = A^*^b+(*I*−A^*^A)*y*, where *y*∈*R*^*k*^ is arbitrary. The following will demonstrate that *G*^*T*^w^*^ is a solution to problem 1, where w^*^ is the general solution to Optimization problem 2:


$$\begin{array}{l} G^{T}w^{*}=G^{T}\left(A^{*}b+\left(I-A^{*}A\right)y\right)\\ =G^{T}A^{*}b+\left(G^{T}-G^{T}A^{*}A\right)y\\ =G^{T}{G^{*}}^{T}G^{*}b+\left(G^{T}-G^{T}{G^{*}}^{T}G^{*}GG^{T}\right)y\\ =G^{*}b+\left(G^{T}-G^{*}GG^{T}\right)y\\ =G^{*}b+\left(G^{T}-G^{T}{G^{*}}^{T}G^{T}\right)y\\ =G^{*}b+\left(G^{T}-G^{T}\right)y=G^{*}b \end{array}$$

In summary, for the generic solution w^*^ of Optimization problem 2, we have *G*^*T*^w^*^ = G^*^b, and G^*^b is the unique solution to Optimization problem 1, then solving Optimization problem 1 can be equivalently converted to solving Optimization problem 2.

To facilitate the discussion of the association between the optimization problem of finding the optimal descent direction in Désidéri (2012) and ours, we first introduce that optimization problem of MGDA in Equation (20) and the definition of Pareto-stationary point in Definition 1. For the optimization problem of MGDA, we chose standard normalization to better balance, geometric properties and theoretical analysis. Optimization problem in Equation (20) will be referred to as Optimization problem 3 in the following discussion.


(20) $$\begin{array}{l} {argmin}_{g}\;||g|{|}_{2}^{2}\;,\\ s.t.\left\{g|g=\overset{k}{\underset{i=1}{\sum }}c_{i}\frac{g_{i}}{||g_{i}|{|}_{2}},\;c_{i}\geq 0\left(i=1,\cdots \;,k\right),\;\overset{k}{\underset{i=1}{\sum }}c_{i}=1\right\}. \end{array}$$

** Definition 1**. *Let* *x*_0_ *be a point at the center of an open ball in the feasible domain* Ω, *k* *smooth objective functions* *J*_*i*_(*x*)_*i* = 1, ⋯ , *k*_ *with* *g*_*i*_ = ∇_*x*_*J*_*i*_(*x*_0_) *being the gradient. A point* *x*_0_ *is said to be Pareto stable if there exists a convex combination of gradient vectors* {_*g*_*i*_}*i* = 1, ⋯ , *k*_ *equal to zero:*


(21) $$\begin{array}{l} \overset{k}{\underset{i=1}{\sum }}c_{i}g_{i}=0,c_{i}\geq 0\left(i=1,\cdots \;,k\right),\overset{k}{\underset{i=1}{\sum }}c_{i}=1. \end{array}$$

Under certain conditions, the solution to Optimisation Problem 3 is in the same direction as Optimisation Problem 1, and I will state and prove this proposition below.

** Proposition 3**. *The solution to Optimisation Problem 3 is in the same direction as Optimisation Problem 1, if the solution* $g^{*}=\sum_{i=1}^{k}c_{i}^{*}\frac{g_{i}}{||g_{i}|{|}_{2}}$ *of Optimization problem 3 has* $c_{i}^{*}>0\left(i=1,\cdots \;,k\right)$ *and* g^*^≠0.

Proof. Let $g^{*}=\sum_{i=1}^{k}c_{i}^{*}\frac{g_{i}}{||g_{i}|{|}_{2}}$ is the solution of Optimization problem 3 with $c_{i}^{*}>0\left(i=1,\cdots \;,k\right)$, $\overset{k}{\underset{i=1}{\sum }}c_{i}^{*}=1$. Since $c_{i}^{*}>0\left(i=1,\cdots \;,k\right)$, none of the inequality constraints is saturated. Consequently, g^*^ is also a optimal solution to the following optimization problem:


(22) $$\begin{array}{l} {argmin}_{g}\;||g|{|}_{2}^{2}\;,\\ s.t.\left\{g|g=\overset{k}{\underset{i=1}{\sum }}c_{i}\frac{g_{i}}{||g_{i}|{|}_{2}},\;\overset{k}{\underset{i=1}{\sum }}c_{i}=1\right\}. \end{array}$$

Consequently, using the vector *c*∈*R*^*k*^ as the finite-dimensional variable and $c^{*}={\left(c_{1}^{*},\cdots \;,c_{k}^{*}\right)}^{T}$. The Lagrangian writes as $L\left(c,\lambda \right)=||\left(\frac{g_{1}}{||g_{1}|{|}_{2}},\cdots \;,\frac{g_{k}}{||g_{k}|{|}_{2}}\right)c|{|}_{2}^{2}+\lambda \left(\overset{k}{\underset{i=1}{\sum }}c_{i}-1\right)$, and for all indices *i*, calculate the partial derivatives for *c*_*i*_:


(23) $$\begin{array}{l} \frac{\partial L}{\partial c_{i}}=\frac{\partial ||\left(\frac{g_{1}}{||g_{1}|{|}_{2}},\cdots \;,\frac{g_{k}}{||g_{k}|{|}_{2}}\right)c|{|}_{2}^{2}}{\partial c_{i}}+\lambda =2\langle \frac{g_{i}}{||g_{i}|{|}_{2}},\overset{k}{\underset{i=1}{\sum }}\frac{g_{i}}{||g_{i}|{|}_{2}}c_{i}\rangle +\lambda . \end{array}$$

Since the optimality condition, $\frac{\partial L}{\partial c_{i}}=0,\frac{\partial L}{\partial \lambda }=0$, is satisfied at the vector c^*^, we have $\langle g_{i},g^{*}\rangle =-\frac{\lambda }{2}||g_{i}|{|}_{2}\left(i=1,\cdots \;,k\right)$, then there are the following equations:


(24) $$\begin{array}{l} \left(\begin{array}{c} \langle \frac{g_{1}}{||g_{1}|{|}_{2}},g^{*}\rangle \\ \;\cdots \\ \langle \frac{g_{k}}{||g_{k}|{|}_{2}},g^{*}\rangle \end{array}\right)=-\frac{\lambda }{2}\left(\begin{array}{c} \;1\\ \cdots \\ \;1 \end{array}\right) \end{array}$$

From lemma 2.1 in Désidéri (2012), for all *g*_*i*_(*i* = 1, ⋯ , *k*): $\langle g_{i},g^{*}\rangle \geq ||g^{*}|{|}_{2}^{2}$; therefore:$-\frac{\lambda }{2}>0$. Since $c^{**}=-\frac{2}{\lambda }c^{*}$ is the optimal solution to Optimisation Problem 2, it is proved that the solution to Optimisation Problem 3 is in the same direction as Optimisation Problem 1.

Proposition 3 illustrates that Optimization Problem 2 is capable of determining the common descent direction for multiple objectives, effectively resolving the issue of insufficient attacks on multiple models. Additionally, we offer a simple geometric explanation for MGDA.

From previous papers (Chen et al., 2021; Zhao et al., 2021), it is argued that more iterative attacks and larger perturbations can effectively improve the transferability of adversarial samples. As well, studies such as Dong et al. (2018) and Wang and He (2021) propose to increase perturbations by stabilizing the update direction and effectively improving transferability. From the definition of the Pareto-stationary point and Optimization problem 3, the MGDA algorithm stops at the Pareto-stationary point, inapplicable for transferability black-box attacks. Conversely, our approach at the Pareto-stationary point can also attack in a direction that is synthetically effective against multiple objectives.

## 4. Experiments

### 4.1. Experimental setup

#### 4.1.1. Dataset

We conducted experiments on the LFW (Huang et al., 2007) dataset, which is the most widely used benchmark for image face verification, contains 13,233 face images from 5,749 different individuals, and includes faces with a variety of poses, expressions, and illuminations. The unrestricted external data protocol with labels includes 6,000 pairs of faces, of which 3,000 pairs have the same identity and 3,000 pairs have different identities. We performed targeted transferability attacks on the 3,000 pairs that have different identities.

#### 4.1.2. Facial recognition models

For our experiments, we used five surrogate models for the ensemble attack, where the pre-training parameters for the models were obtained from the open-source library in the paper (Wang Q. et al., 2021). The number of unknown black-box models tested was twenty, with ten normally trained models and ten defensive models, and the pre-training parameters for the models were obtained from the open-source library of RobFR (Yang et al., 2020). We tabulate the full model information in Table 1, which includes the name of the models, as well as the best threshold of discrimination (Threshold) on the LFW dataset and the accuracy (Acc) concerning it. Among all the defense models, the top three defense models adopt the defense transform methods (BR, Xu et al., 2017; RP, Xie et al., 2018; JPEG, Dziugaite et al., 2016), and the next seven are adversarially trained models.

**Table 1:** The models' information includes sequence number, name, the best discriminant threshold (threshold), and the accuracy (Acc) corresponding to the threshold on the LFW dataset.

**Normal black-box models**				**Defense black-box models**				**Surrogate models**			
**No**	**Model's name**	**Threshold**	**Acc**	**No**	**Model's name**	**Threshold**	**Acc**	**No**	**Model's name**	**Threshold**	**Acc**
M-1	FaceNet-casia	0.27	0.91	M-11	IR50-Softmax-BR	0.33	0.99	M-21	IR_SE_50_MS1M	0.24	0.96
M-2	FaceNet-VGGFace2	0.28	0.95	M-12	IR50-Softmax-RP	0.35	0.99	M-22	IR_50_Asia	0.22	0.92
M-3	CASIA-SphereFace	0.43	0.97	M-13	IR50-Softmax-JPEG	0.34	0.99	M-23	IR_50_Ms1m	0.17	0.95
M-4	CosFace	0.24	0.98	M-14	IR50-PGDSoftmax	0.3	0.91	M-24	IR_101_Ms1m	0.22	0.86
M-5	IR50-SphereFace	0.36	0.99	M-15	IR50-TradesSoftmax	0.33	0.91	M-25	IR_152_MS1M	0.16	0.95
M-6	Mobilenet	0.15	0.99	M-16	IR50-TradesCosFace	0.63	0.91				
M-7	IR50-Softmax	0.34	0.99	M-17	IR50-PGDCosFace	0.23	0.86				
M-8	CASIA-Softmax	0.32	0.98	M-18	IR50-PGDAm	0.41	0.85				
M-9	CASIA-Am	0.46	0.98	M-19	IR50-PGDArcFace	0.37	0.87				
M-10	SphereFace	0.35	0.98	M-20	IR50-TradesArcFace	0.85	0.95				

1 − 10 are normal training models, 11 − 13 are models with input transformation defense methods, and 14 − 20 are adversarial training models, and 21 − 25 are surrogate models.

#### 4.1.3. Baselines

The baseline methods to compare are the extensively studied gradient-based attack method, including I-FGSM (Madry et al., 2018), TI-FGSM (Dong et al., 2019), DI-FGSM (Xie et al., 2019), DI-TI-MI-FGSM, and SI-DI-FGSM (Lin et al., 2019). For the baseline approach, we used SVRE and Ens as ensemble methods, where Ens is a uniform fusion of the losses from different models.

#### 4.1.4. Evaluation metric

Given an attack method $A_{\epsilon }\left(x,x_{target}\right)$ under the *L*_*inf*_ norm, an adversarial example $x^{adv}=A_{\epsilon }\left(x,x^{target}\right)$ is generated for the input *x* and the target image *x*^*target*^. Following the previous face recognition (Wang Q. et al., 2021) and targeted attack settings (Yang et al., 2020; Zhao et al., 2021), the evaluation metric is the attack success rate (Asr) on the face recognition model *F*_*i*_(*x*), defined as follows.


(25) $$\begin{array}{l} Asr\left(F_{i},A_{\varepsilon }\right)=\frac{1}{\text{N}}\overset{N}{\underset{j=1}{\sum }}I\left(\cos\left(F_{i}\left(x_{j}^{target}\right),F_{i}\left(x_{i}^{adv}\right)\right)>\delta_{i}\right),\;x_{i}^{adv}\\ =A_{\varepsilon }\left(x_{j},x_{j}^{target}\right) \end{array}$$

where ${\left(x_{i},x_{i}^{target}\right)}_{i=1}^{N}$ is the paired test set, *I*(·) is the indicator function, and δ_*i*_ is the best threshold corresponding to *F*_*i*_.

#### 4.1.5. Hyper-parameters

Following previous work (Dong et al., 2018; Lin et al., 2019; Xie et al., 2019; Yang et al., 2020), we set the maximum perturbation to ϵ = 8, the range of pixel values for each image to [0, 255], the number of iterations was 20, and the step size was α = 0.6. For MIM, we set the decay factor μ = 1. For TIM, we used a Gaussian kernel of size 7 × 7. For DIM, the transition probability *p* was set to 0.8. For SIM, we set the number of copies *m* = 4. For SVRE, we set the internal update frequency *M* to four times the number of ensemble models, the internal step size β is set 0.6 and the internal decay factor μ_2_ is set to 1.0. For RGEA, we set the step size α = 1, since our method removes the sign function, resulting in much smaller perturbations at each iteration than Ens. For fairness, we also verified the disturbance distance $l_{2}\left(x\right)=||x|{|}_{2}/\sqrt{d},x\in R^{d}$ of RobFR (Yang et al., 2020) in Table 2.

**Table 2:** Perturbation distance (*l*_2_) for the adversarial examples and success rate (%) of attacks on the normal model for Ens, SVRE, Ens-*l*_2_ and RGEA, where the adversarial examples were all crafted on the surrogate models.

**Base**	**Attack**	**M-1**	**M-2**	**M-3**	**M-4**	**M-5**	**M-6**	**M-7**	**M-8**	**M-9**	**M-10**	**Average**	***l*_2_**
I-FGSM	Ens	49.07	38.73	49.80	59.23	70.73	57.93	71.87	70.17	68.57	65.07	60.12	5.21
	SVRE	14.50	7.43	17.30	15.17	22.17	10.57	21.07	19.33	18.53	19.07	16.51	**3.43**
	Ens-*l*_2_	52.87	42.23	53.70	61.57	73.20	60.73	74.23	73.47	70.57	68.10	63.07	4.24
	RGEA	**57.70**	**45.23**	**57.63**	**66.77**	**76.93**	**65.23**	**78.93**	**79.33**	**77.30**	**73.20**	**67.83**	4.44
DI-FGSM	Ens	50.47	41.10	51.83	61.70	71.93	62.33	73.33	72.50	70.60	67.47	62.33	4.47
	SVRE	18.57	10.37	23.03	22.53	30.87	18.03	29.50	28.37	25.97	26.50	23.37	**3.11**
	Ens-*l*_2_	57.03	47.70	58.80	67.33	77.57	68.27	78.13	77.97	75.43	72.40	68.06	4.02
	RGEA	**64.43**	**54.40**	**64.87**	**74.17**	**81.97**	**74.03**	**83.70**	**84.27**	**82.90**	**79.07**	**74.38**	4.39
DI-TI-FGSM	Ens	52.07	43.60	53.63	64.07	73.43	64.80	74.93	73.70	72.47	69.33	64.20	4.59
	SVRE	21.67	12.67	26.13	25.77	35.53	21.60	33.90	32.13	29.70	30.63	26.95	**3.23**
	Ens-*l*_2_	58.53	49.23	59.57	69.00	77.83	69.77	78.93	78.60	76.80	73.90	69.22	4.11
	RGEA	**65.17**	**56.00**	**65.67**	**75.43**	**82.70**	**74.80**	**84.60**	**84.77**	**84.03**	**79.63**	**75.28**	4.44
DI-TI-MI-FGSM	Ens	51.70	45.20	52.97	66.77	73.30	66.83	76.13	75.60	75.03	71.47	65.50	7.00
	SVRE	64.73	**59.23**	65.83	77.20	81.97	77.20	85.00	84.20	82.97	79.77	75.81	7.25
	Ens-*l*_2_	58.30	51.20	59.12	72.14	78.23	72.15	80.20	80.12	80.41	75.12	70.69	**4.56**
	RGEA	**67.77**	58.43	**67.50**	**77.40**	**83.43**	**76.56**	**86.00**	**86.20**	**85.20**	**80.86**	**76.93**	4.83
SI-DI-FGSM	Ens	59.57	52.00	58.43	72.40	77.97	73.33	80.40	80.10	80.47	76.40	71.11	5.55
	SVRE	36.50	27.53	42.50	47.50	57.40	44.70	55.87	54.57	51.43	51.30	46.93	**3.73**
	Ens-*l*_2_	62.70	55.03	61.90	73.83	79.37	74.33	81.73	81.93	81.17	77.77	72.98	4.32
	RGEA	**67.67**	**59.43**	**66.10**	**77.77**	**82.30**	**77.73**	**84.93**	**85.33**	**85.33**	**81.40**	**76.80**	4.88

The bold values are the best.

### 4.2. Ablation study

The ablation study is conducted to verify the benefits of RGEA and to determine the effects of key parameters. Specifically, we first tested the effect of parameters in existing methods on RGEA and then investigated the effectiveness of Optimization Problem 2.

#### 4.2.1. Effect of parameters

In this section, we explore the impact of *p* in DI and μ in MI for RGEA using five normally trained models (M-6, M-7, M-8, M-9, and M-10).

**Figure 3:**
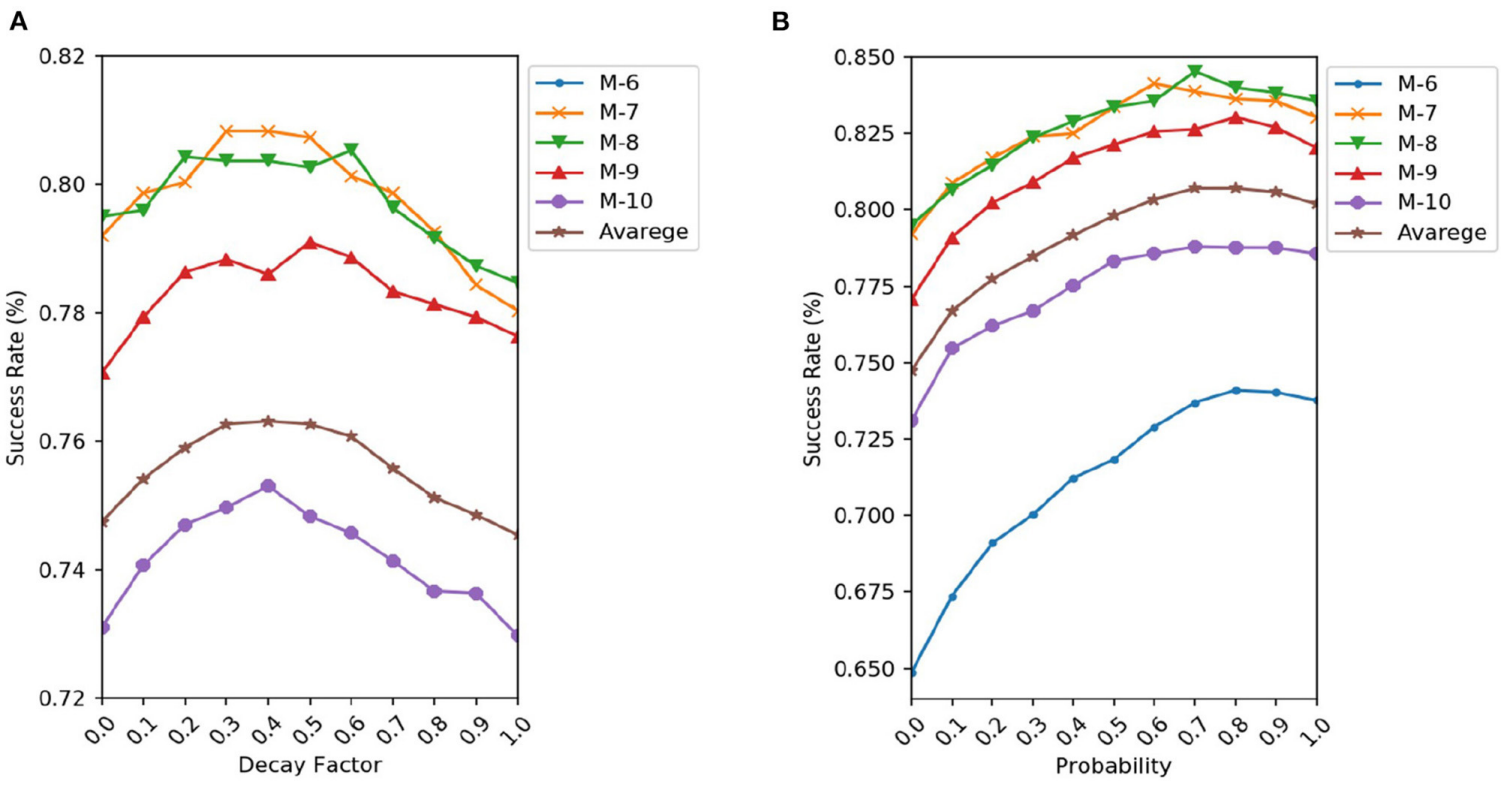
Success rate of RGEA-MI-FGSM **(A)** and RGEA-DI-FGSM **(B)** when the corresponding parameters are changed.

**On the decay factor** **μ** **in RGEA-MI-FGSM:** We investigate the influence of the decay factor μ of the RGEA-MI-FGSM on the success rate. We combine the MI-FGSM attack with the RGEA, and the decay factor μ has a granularity of 0.1 and runs from 0 to 1. RGEA-MI-FGSM degrades to the RGEA-I-FGSM attack method if μ = 0. Figure 3A displays several networks' success rates and their average values. In contrast to the experimental results in Dong et al. (2018), we observe that the attack success rate of RGEA-MI-FGSM increases as μ rises and reaches a maximum around μ = 0.4, after which the success rate significantly decreases. It is possible that too large μ destroys the optimal direction of descent for the current calculation, resulting in a significant decrease in transferability. In the following experiments, we will set μ = 0.4 for RGEA.

**On the probability** **p** **in RGEA-DI-FGSM:** We then study the influence of the transformation probability *p* on the success rates under black-box settings. The transformation probability *p* varies from 0 to 1 and RGEA-DI-FGSM degrades to RGEA-I-FGSM if *p* = 0. We show the success rates on various networks and their average values in Figure 3B. Similar to the experimental results in the paper (Xie et al., 2019), the black-box success rate of RGEA-DI-FGSM is higher as *p* increases. Furthermore, the black-box success rate can be significantly increased if *p* is small, i.e., if only a small number of transformed inputs are used. In the following experiments, we will set *p* = 0.8 for RGEA.

#### 4.2.2. Effect of optimization problem 2

In this section, ablation experiments are conducted to evaluate the efficacy of Optimization Problem 2. Specifically, we drop Optimization Problem 2 for finding the optimal descent direction in RGEA, but we keep the normalization approach in equation (18) and name it as Ens-*l*_2_. Then, on the normally trained model, compared the transferability of RGEA and Ens-*l*_2_.

In Section 4.3.1, the findings reveal that RGEA outperforms Ens-*l*_2_ in all experiments, where on DI-FGSM, DI-TI-FGSM, DI-TI-MI-FGSM, and SI-DI-FGSM, the average success of RGEA is increased by 6.32, 6.06, 6.24, and 3.82%, respectively. The results show that Optimization Problem 2 in RGEA can effectively exploit the complex inter-model dependencies and improve transferability.

**Figure 4:**
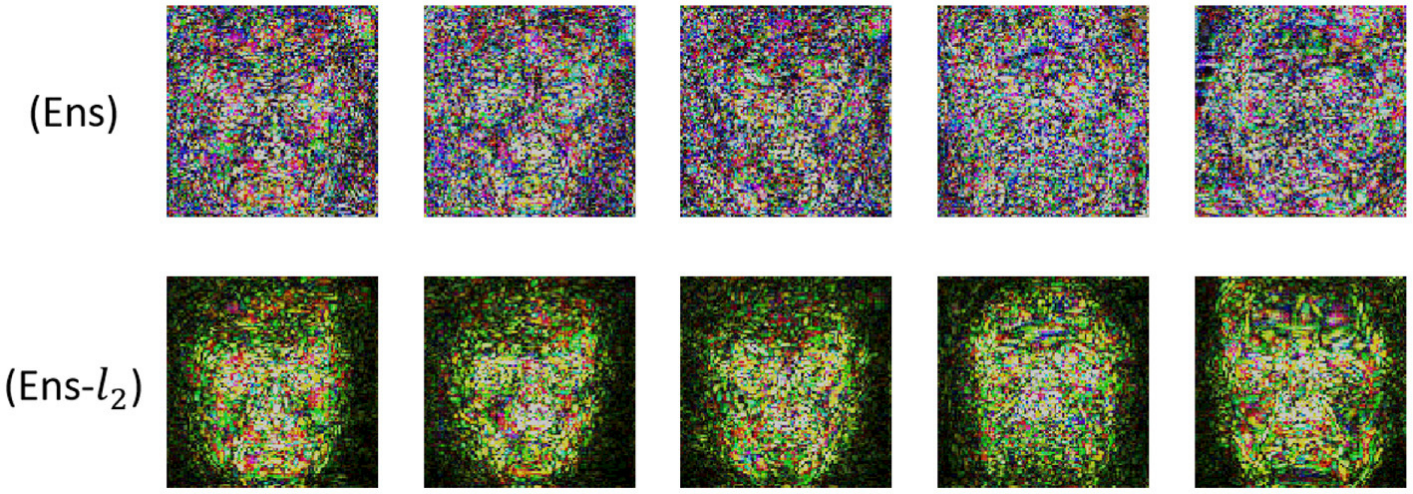
The adversarial perturbation noise images for Ens and Ens-*l*_2_ with I-FGSM.

Furthermore, we found that Ens-*l*_2_ outperformed Ens in all experiments. It may be that targeted transferability relies on activating target semantic features, yet this is different from non-targeted transferability, which attacks the activated features. The result is that targeted transferability attacks need a more precise attack direction during each iteration. For this observation, we further visualize the adversarial perturbation noise images of Ens and Ens-*l*_2_. In Figure 4, we can see that the adversarial perturbation noise image generated by Ens-*l*_2_ has more semantical information about the target face.

### 4.3. Comparisons with state-of-the-art methods

#### 4.3.1. Attack normally trained models

We first compared the transferability capabilities on normal training models. Specifically, adversarial samples were generated on multiple surrogate models by Ens, SVRE and RGEA combined with various basic methods and then tested for *l*_2_ perturbation distance and the attack success on normal training models. The attack performance of the normal training models is displayed in Table 2.

In general, RGEA performed better than Ens and SVRE in almost experiments. Especially in DI-FGSM and DI-TI-FGSM, RGEA is higher than Ens by 12.05 and 11.08%, and 1.12% higher than SVRE in TI-DI-MI-FGSM. The disturbances of RGEA are smaller than Ens, which indicates that rather than just increasing the perturbation, the RGEA improved transferability from successfully utilizing the complex dependencies among the models. Furthermore, we find that compared with Ens, RGEA-I-FGSM combined with DI shows fewer changes in disturbances, which indicates that RGEA can effectively alleviate the instability problems caused by DI.

For SVRE, except DI-TI-MI-FGSM, it is significantly lower than Ens and RGEA in targeted transferability black-box attack tests. Regarding this phenomenon, we attribute it to three aspects: 1) SVRE embeds an inner loop in the iterative attack, where the inner loop iteratively randomly selects models to compute gradients for internal updating to correct the average gradient in the iterative attack. However, SVRE chooses the inner loop's last update direction as the iterative attack's update direction, and the update step size of both is the same large. This causes the iterative attack's update direction loses the current iteration point's local information, which destroys the effectiveness of the iterative attack method. Meanwhile, with the same normalization operation and step size, the perturbation of SVRE is much smaller than Ens, which strongly supports the view. 2) For MI, SVRE also uses the momentum method in the inner loop and updates the iterative attack's momentum using the inner loop's momentum. This skillfully overcomes the ineffective forward-looking of the momentum method and also stabilizes the update direction. This perspective is supported by the fact that for DI-TI-MI-FGSM, the SVRE outperforms Ens by 10.31 and the perturbation up to 7.25. 3) SVRE only considers non-target attacks, however target attacks are more sensitive to the precision of the update direction compared to non-target attacks.

#### 4.3.2. Attack advanced defense models

To more comprehensively verify the effectiveness of RGEA, experiments were conducted on models with defensive capabilities. Specifically, Ens, SVRE and RGEA were combined with various basic methods to generate adversarial samples and then tested on input transformations and adversarially trained defense models.

For the defense model with input transformation, shown in Table 3, the results indicate a significant advantage of RGEA over Ens and SVRE for all tests. In particular, the average attack success rate of RGEA is 11.03% higher than Ens on DI-FGSM, and is 0.71% higher than SVRE on DI-TI-MI-FGSM. In addition, the results of the white-box attack are shown in Table 3, RGEA improves the performance of the attack in most cases. This indicates that RGEA exploits the dependencies among the models while not destroying the attack capability.

**Table 3:** The black-box attack success rates (%) against three models with the defense transform methods (BR, Xu et al., 2017; RP, Xie et al., 2018; and JPEG, Dziugaite et al., 2016).

**Base**	**Attack**	**Surrogate models**						**Defense black-box models**			
		**M-21**	**M-22**	**M-23**	**M-24**	**M-25**	**Average**	**M-11**	**M-12**	**M-13**	**Average**
I-FGSM	Ens	100.00	**99.70**	**99.97**	100.00	99.97	99.93	71.77	65.97	67.6	68.44
	SVRE	73.10	43.20	43.53	51.27	55.50	53.32	20.90	16.53	15.97	17.80
	RGEA	**100.00**	99.60	99.80	**100.00**	**100.00**	**99.88**	**78.57**	**74.5**	**75.73**	**76.27**
DI-FGSM	Ens	**99.97**	93.37	95.60	99.77	99.73	97.69	73.4	69.8	70.03	71.08
	SVRE	74.97	39.67	34.63	67.70	52.23	53.84	29.60	25.50	24.27	26.46
	RGEA	99.93	**96.20**	**97.93**	**100.00**	**99.83**	**98.78**	**83.37**	**81.27**	**81.7**	**82.11**
DI-TI-FGSM	Ens	99.97	93.37	96.40	99.70	99.77	97.84	74.57	71.27	71.87	72.57
	SVRE	80.97	45.70	37.27	66.27	51.07	56.25	33.83	30.80	29.00	31.21
	RGEA	**99.97**	**95.83**	**97.97**	**100.00**	**99.87**	**98.73**	**84.33**	**82.5**	**82.63**	**83.16**
DI-TI-MI-FGSM	Ens	99.97	90.80	94.90	99.87	99.67	97.04	76.3	73.4	74.73	74.81
	SVRE	100.00	96.40	98.63	100.00	99.90	98.99	85.00	82.97	83.47	83.81
	RGEA	**100.00**	**98.40**	**99.16**	**100.00**	**99.90**	**99.49**	**85.87**	**83.67**	**84.03**	**84.52**
SI-DI-FGSM	Ens	99.97	93.03	97.63	**99.93**	**99.90**	98.09	80.27	78.17	78.37	78.93
	SVRE	91.60	63.77	66.00	88.10	78.73	77.64	55.90	53.47	51.73	53.70
	RGEA	**100.00**	**94.20**	**97.93**	99.57	99.73	**98.29**	**84.6**	**83.03**	**82.9**	**83.51**

The adversarial examples are generated on the surrogate models, i.e., M-21, M-22, M-23, M-24, and M-25. The bold values are the best.

In Table 4, RGEA exhibits gains in all tests for the adversarial training model, except DI-TI-MI-FGSM. In addition, the transferability impacts of all approaches are all below 23%. It suggests that there is a significant amount of space for advancement in RGEA for target transferability attacks on adversarial training models and deserves further investigation.

**Table 4:** The black-box attack success rates (%) against seven adversarially trained models.

**Base**	**Attack**	**M-14**	**M-15**	**M-16**	**M-17**	**M-18**	**M-19**	**M-20**	**Average**
I-FGSM	Ens	9.27	10.40	17.33	13.37	13.80	11.73	12.63	12.65
	SVRE	8.03	8.87	11.90	11.93	12.80	10.37	9.70	10.51
	RGEA	**9.60**	**10.83**	**19.37**	**13.70**	**13.77**	**12.03**	**13.73**	**13.29**
DI-FGSM	Ens	9.23	10.60	17.77	13.33	13.87	11.53	12.73	12.72
	SVRE	7.87	9.00	12.27	11.97	12.80	10.30	9.67	10.55
	RGEA	**9.97**	**11.47**	**21.00**	**13.87**	**14.20**	**12.07**	**14.93**	**13.93**
DI-TI-FGSM	Ens	9.50	10.90	18.43	13.47	14.00	11.73	13.30	13.05
	SVRE	8.10	9.30	12.60	12.23	13.13	10.70	10.17	10.89
	RGEA	**10.03**	**11.77**	**21.33**	**14.13**	**14.17**	**12.27**	**15.40**	**14.16**
DI-TI-MI-FGSM	Ens	10.20	11.63	19.67	14.10	14.40	12.33	14.27	13.80
	SVRE	**10.87**	**12.53**	**22.53**	**14.70**	**14.97**	**12.90**	**16.30**	**14.97**
	RGEA	10.26	12.00	21.93	14.20	14.43	12.33	15.67	14.40
SI-DI-FGSM	Ens	10.27	12.00	20.93	14.07	14.67	12.50	15.23	14.24
	SVRE	8.87	10.17	15.30	13.10	13.57	11.13	11.73	11.98
	RGEA	**10.57**	**12.30**	**22.43**	**14.37**	**14.87**	**12.70**	**16.33**	**14.80**

The bold values are the best.

Additionally, we conduct a comparative analysis referring to Tables 3, 4. The input-transformed defense model exhibited worse defense compared to the adversarial training model. Probably, the input transformation only partially disrupts the adversarial perturbation, but it does not change the recognition pattern of the model. Contrarily, adversarial training makes the model acquire a completely different recognition pattern compared to the normally trained model.

## 5. Discussion and conclusion

In this paper, we propose the Relational Graphs Ensemble Attack (RGEA) to enhance the transferability of black-box attacks. Specifically, We find that facial recognition models, compared to classification models, have more complex correlations. This inspired us to exploit the complex dependencies among models to improve the transferability of the generated adversarial samples. To this end, we designed a suboptimization problem based on a multi-model relationship graph to obtain a more transferable descent direction. Extensive experiments show that RGEA significantly improves the transferability of almost baseline methods in a black box environment.

For the transferability black-box attacks, we provide a new perspective to enhance the adversarial transferability, i.e., to facilitate the transferability of adversarial samples by efficiently extracting the complex dependencies among models by graphs. Additionally, our experimental results show that: for targeted transferability attacks on adversarially trained models, there is still significant room for improvement in RGEA and existing methods. We will continue to develop more effective methods to extract complex dependencies among models to overcome this challenge in the future.

## Data availability statement

The original contributions presented in the study are included in the article/supplementary material, further inquiries can be directed to the corresponding author.

## Author contributions

CL: conceptualization, methodology, and software. JP and NJ: validation. JP and CL: formal analysis and writing-original draft preparation. HW: investigation and visualization. ZW and JP: resources, writing-review, editing, and supervision. JP: data curation and project administration. ZW: funding acquisition. All authors have read and agreed to the published version of the manuscript.
